# Toughening and polymerization stress control in composites using thiourethane-treated fillers

**DOI:** 10.1038/s41598-021-87151-9

**Published:** 2021-04-07

**Authors:** Ana Paula Piovezan Fugolin, Ana Rosa Costa, Lourenco Correr-Sobrinho, R. Crystal Chaw, Steven Lewis, Jack Liborio Ferracane, Carmem Silvia Pfeifer

**Affiliations:** 1 Division of Biomaterials and Biomechanics, Department of Restorative Dentistry, Oregon Health & Science University, Portland, OR USA; 2 Division of Dental Materials, Department of Restorative Dentistry, Piracicaba Dental School—UNICAMP, Piracicaba, SP Brazil; 3 Advanced Light Microscopy Core, Department of Neurology, Oregon Health & Science University, Portland, OR USA

**Keywords:** Medical research, Chemistry, Engineering, Materials science

## Abstract

Filler particle functionalization with thiourethane oligomers has been shown to increase fracture toughness and decrease polymerization stress in dental composites, though the mechanism is poorly understood. The aim of this study was to systematically characterize the effect of the type of filler surface functionalization on the physicochemical properties of experimental resin composites containing fillers of different size and volume fraction. Barium glass fillers (1, 3 and 10 µm) were functionalized with 2 wt% thiourethane-silane (TU-Sil) synthesized de novo and characterized by thermogravimetric analysis. Fillers treated with 3-(Trimethoxysilyl)propyl methacrylate (MA-Sil) and with no surface treatment (No-Sil) served as controls. Fillers (50, 60 and 70 wt%) were incorporated into BisGMA-UDMA-TEGDMA (5:3:2) containing camphorquinone/ethyl-4-dimethylaminobenzoate (0.2/0.8 wt%) and 0.2 wt% di-tert-butyl hydroxytoluene. The functionalized particles were characterized by thermogravimetric analysis and a representative group was tagged with methacrylated rhodamine B and analyzed by confocal laser scanning microscopy. Polymerization kinetics were assessed by near-IR spectroscopy. Polymerization stress was tested in a cantilever system, and fracture toughness was assessed with single edge-notched beams. Fracture surfaces were characterized by SEM. Data were analyzed with ANOVA/Tukey's test (α = 0.05). The grafting of thiourethane oligomer onto the surface of the filler particles led to reductions in polymerization stress ranging between 41 and 54%, without affecting the viscosity of the composite. Fracture toughness increased on average by 35% for composites with the experimental fillers compared with the traditional methacrylate-silanized groups. SEM and confocal analyses demonstrate that the coverage of the filler surface was not homogeneous and varied with the size of the filler. The average silane layer for the 1 µm particle functionalized with the thiourethane was 206 nm, much thicker than reported for traditional silanes. In summary, this study systematically characterized the silane layer and established structure–property relationships for methacrylate and thiourethane silane-containing materials. The results demonstrate that significant stress reductions and fracture toughness increases are obtained by judiciously tailoring the organic–inorganic interface in dental composites.

## Introduction

Satisfactory mechanical properties and ability to mimic the dental substrate make resin composites the most popular choice for direct dental restorations. However, fifty percent of all resin-based restorations fail in less than 10 years, which inevitably causes unnecessary removal of additional sound tooth structure and pain and discomfort to the patient, in addition to costing millions of dollars annually^1^. The poor quality of the bond and seal at the restoration margin due to the stress generated during the polymerization reaction is implicated as one of the factors leading to the replacement of dental composite restorations^2,3^. In addition, fracture is also indicated as one of the primary reasons for the limited clinical lifespan of composite restorations^1^. Thus, efforts have been focused on modifying the composition of dental composite restoratives to address these shortcomings.

One of the latest developments in composite formulation has been the introduction of toughening, stress-reducing thiourethane oligomer additives. These are high molecular weight species, obtained via a facile click reaction chemistry that produces loosely crosslinked pre-networks bearing pendant thiol functionalities. When added to methacrylate monomer mixtures, the pendant thiols undergo chain-transfer reactions with the vinyl free radicals, which delays gelation/vitrification and leads to an overall improvement in conversion and a decrease in polymerization stress^4–6^. In general, these additives have been successful in reducing polymerization stress without jeopardizing elastic modulus and degree of conversion of the final polymer. However, a common effect of the addition of pre-polymerized oligomers to the composite’s resin matrix is an increase in the resin viscosity, which can potentially limit the amount of inorganic filler that can be incorporated, as well as compromise overall handling characteristics. One strategy to overcome this limitation is to graft thiourethane oligomers to the surface of filler particles. One recent publication using this approach demonstrated up to 35% reduction in polymerization stress for the resultant composite, accompanied by significantly improved mechanical properties^7^. This is especially promising because it may allow for more homogenous distribution of the thiourethane oligomer into the material, while minimizing the impact on the final viscosity of the composite^7^.

Despite the demonstrated benefits of the use of thiourethane oligomers as additives to the resin matrix^5,6^ or to the filler surface^8^, the mechanism for stress reduction and toughening effects is not completely understood. For instance, in separate studies, the net concentrations of thiourethanes added to the matrix, i.e., approximately 20%^5,6^, or to the filler surface, i.e. roughly 6%^8^, were markedly different, yet the levels of stress reduction and toughening were similar. Chain-transfer reactions alone cannot explain the similarities, since the concentration of pendant SH in the filler-based studies was relatively low. One hypothesis is that the formation of low Tg polymer brush structures on the surface of the filler are able to deform to compensate for the change in free volume inherent in the polymerization reaction^9,10^. This strategy targets the interface between filler and matrix, one area where stress concentration is more marked^11^.

To better understand the observed effects and provide a mechanism of action for the thiourethane additive requires a more systematic evaluation that takes into consideration the size and loading of fillers. Therefore, the aim of the present study was to assess the effect of particle size and loading, as well as the type of filler surface treatment, on polymerization stress development and fracture toughness of experimental resin composites. Polymerization kinetics and film thickness were also evaluated to ensure that producing a formulation with excellent shrinkage stress and toughness properties did not compromise the clinically relevant characteristics of curing efficiency and handling. The tested hypothesis was that the reduction in polymerization stress and increase in fracture toughness expected with the thiourethane-treated particles will be potentiated as the overall content of thiourethane increases.

## Materials and methods

### Thiourethane synthesis and functionalization of filler particles

All chemicals were sourced from Sigma-Aldrich (Milwaukee, WI, USA) and used without further purification, unless otherwise noted. The thiourethane (TU) oligomer was obtained by a click reaction in solution of pentaerythritol tetra-3-mercaptopropionate (PETMP), 1,3-bis(1-isocyanato-1-methylethyl)benzene (BDI), and 3-(triethoxysilyl)propyl isocyanate (2.5:1:1 mol, respectively), as previously described in detail^6^. The purified oligomers were characterized by mid-IR (by the disappearance of the isocyanate peak at 2270 cm^-1^) and NMR spectroscopy (appearance of resonance signals at 3.70 ppm).

Filler particles were functionalized with the TU silane described above or 3-(Trimethoxysilyl)propyl methacrylate, which were mixed at 2 wt% in an ethanol:millipore water solution (80:20 vol%) acidified by glacial acetic acid (pH ~ 4.5). This concentration was determined in a previous study in order to ensure full coverage of the particles^12^. Barium-alumino silicate particles (average size of 1.0 ± 0.2, 3.0 ± 1.0 and 10.0 ± 2.0 µm, Specialty Glass, Oldsmar, FL) were dispersed into the solution, agitated for 24 h and then filtered, washed in hexanes, and dried for 4 days at 37 °C. The selected sizes represent a range of the larger size of particles used in hybrid composites. Particles were characterized with thermogravimetric analysis (Discovery TGA55, TA Instruments—Waters LLC, New Castle, DE). Approximately 15 mg of filler was placed in a platinum pan and subjected to a heat ramp (50 to 850 °C, 10 °C/min). Percent mass loss was recorded as a function of temperature and all samples were tested in triplicate. Results were reported as the average total mass loss (%).

### Experimental groups

**Figure 1:**
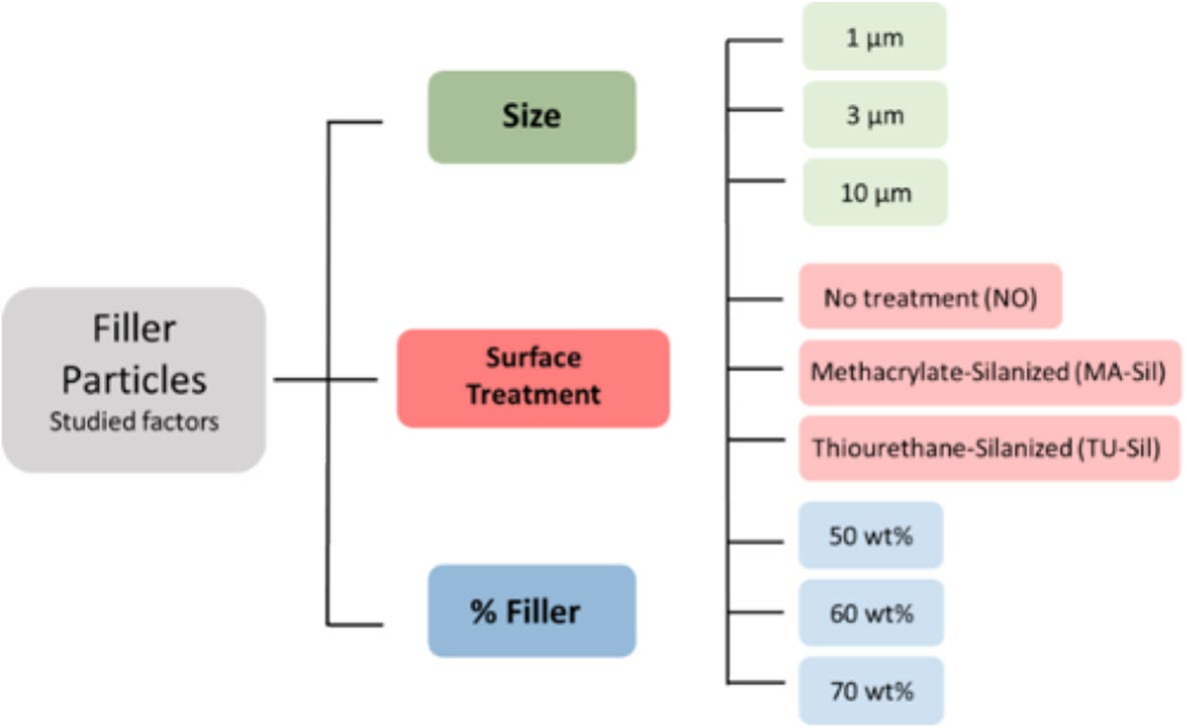
Fluxogram showing the variables studied—size, functionalization and amount of filler particles—and depicting the twenty-seven experimental groups.

Experimental resin composites containing 50, 60 or 70 wt% (26.8, 35.5, and 46.1 vol%, respectively) of the three different sizes of thiourethane-functionalized, methacrylate-silanized or untreated filler particles were produced by mixing the fillers into an organic resin matrix composed of 50 wt% BisGMA (Bisphenol A diglycidyl dimethacrylate), 30 wt% UDMA (urethane dimethacrylate), and 20 wt% TEGDMA (triethylene glycol dimethacrylate). The photoinitiator system was composed by 0.2 wt% camphorquinone, 0.8 wt% EDMAB (ethyl 4-dimethylaminobenzoate), and 0.2 wt% inhibitor BHT (2,6-di-tert-butyl-4-methylphenol). The twenty seven experimental groups are summarized in Fig. 1. All photocuring procedures were carried out using the DEMI Plus, with a 8 mm light guide (Kerr Dental, Orange, CA, USA) and 550 mW/cm^2^ radiant exitance as checked daily using a laser power meter (PM5200 Power Max, PM3 sensor, Molectron, Portland, OR, USA).

### Polymerization stress

The stress of polymerization was assessed using a single cantilever system—Bioman, consisting of a specimen sandwiched between a fixed fused silica slide and a steel piston connected to a load cell^13^. The piston surface (5 mm in diameter) was treated with a metal primer (Z-Prime Plus, Bisco Inc., Schaumburg, IL, USA) and the fused silica slide surface was treated with silane (Ceramic Primer, 3 M ESPE, St. Paul, MN, USA). The 0.8-mm gap between the upper piston and the lower silica slide was filled with the composite paste, and the force generated by the shrinkage of the materials was followed in real-time during the photopolymerization (40 s, through the glass, at 550 mW/cm^2^). Data were recorded for 10 min and the final value was used for the calculation of contraction stress (n = 5).

### Fracture toughness

The single-edge notch beam method was used to analyze the fracture toughness, according to ASTM Standard E399-90^14^. Resin composite bars of 5 mm × 2 mm × 25 mm were prepared in split steel molds having a razor blade insert, providing a 2.5 mm long notch at the center and through the thickness of the cured beam. The samples, sandwiched between two glass slides, were irradiated for 60 s on each side (4 overlapping exposures of 20 s each to cover the entire bar length) with the light guide in contact with the glass. After 24 h in dry storage, the fracture toughness test was carried out in three-point bending (20 mm span) in a universal testing machine (MTS Criterion, Eden Prairie, MN, US) at a cross-head speed of 0.5 mm/min (n = 8).

Fracture toughness was calculated according to the following Eq. (1):

1 $${K_{IC}} = \frac{3PL}{{2B{W^{{3 \mathord{\left/ {\vphantom {3 2}} \right. \kern-\nulldelimiterspace} 2}}}}}\left\{ {1.93{{\left( \frac{a}{W} \right)}^{{1 \mathord{\left/ {\vphantom {1 2}} \right. \kern-\nulldelimiterspace} 2}}} - 3.07{{\left( \frac{a}{W} \right)}^{{3 \mathord{\left/ {\vphantom {3 2}} \right. \kern-\nulldelimiterspace} 2}}} + 14.53{{\left( \frac{a}{W} \right)}^{{5 \mathord{\left/ {\vphantom {5 2}} \right. \kern-\nulldelimiterspace} 2}}} - 25.11{{\left( \frac{a}{W} \right)}^{{7 \mathord{\left/ {\vphantom {7 2}} \right. \kern-\nulldelimiterspace} 2}}} + 25.8{{\left( \frac{a}{W} \right)}^{{9 \mathord{\left/ {\vphantom {9 2}} \right. \kern-\nulldelimiterspace} 2}}}} \right\}$$

where: ***P*** is load at fracture (N), ***L*** is the length, ***B*** is the thickness, ***W*** is the width, and ***a*** is the notch length (all dimensions in mm).

### Kinetics of polymerization

Cylindrical samples (6 mm in diameter and 0.8 mm in thickness) were photoactivated through a glass slide (40 s, 1.5 cm of distance between the Demi light guide and glass slide surface, delivering 150 mW/cm^2^ to the surface of the specimen). The polymerization was followed in real time (180 s) using the methacrylate vinyl overtone peak at 6165 cm^−1^ by near-infrared spectroscopy (near-IR—Nicolet 6700, Thermo Electron Corporation, Waltham, MA, US) at 2 scans per spectrum with 4 cm^−1^ resolution (n = 3). The area of the peak was used to calculate degree of conversion (DC) as a function of time, and rate of polymerization^15^. The DC at the maximum rate of polymerization (DC at RP_MAX_) was used as a proxy for the onset of vitrification.

### Handling/film thickness

Specimen preparation for testing the film thickness followed ISO 4049 guidelines, as described previously in the literature^16^. In summary, 0.1 g of the uncured composites was laminated between two Mylar sheets and statically loaded with 2 kg for 60 s. At the end of this time, the load was removed and the samples photocured for 60 s on each side. The thickness of the resin composite films was measured with a digital caliper at five different locations for each sample and the mean of the measurements was calculated as the final thickness (n = 5).

### Space between the particles

The spacing between the filler particles for each experimental group was estimated with Eq. (2), making the broad assumptions of a spherical particulate of uniform size and homogenous distribution of the particles in the resin:

2 $${\mathrm{Ds}}=\frac{[2\times {\mathrm{Dp}}\times (1-{\mathrm{Vp}})]}{3\times {\mathrm{Vp}}},$$

where: **Dp** is the average filler size (in µm) and **Vp** is the filler volume fraction .

### Microscopy analyses

After testing, fracture toughness bars were cleaned in water in an ultrasonic bath for 10 min and mounted on aluminum stubs. Fracture surfaces were coated with approximately a 6 nm layer of gold/palladium (Leica EM ACE600 High Vacuum Sputter Coating). Imaging was carried out under high vacuum, accelerating voltage of 20 kV and working distance around 10 mm (JEOL, JSM-5600 LV, SEM, Japan).

The 1 µm-sized thiourethane functionalized filler particles were tagged with Methacryloxyethyl thiocarbamoyl rhodamine B (PolyFluor 570, Polysciences, Warrington, PA, USA) for confocal imaging. In summary, 2 g of filler were stirred with 0.1 wt% of the methacrylated rhodamine for 24 h in 5 ml of methylene chloride and catalytic amounts of triethylamine. The fillers were washed with acetone, ethanol and water and dried at 37 °C for 4 days. The tagged fillers were incorporated at 10 wt% in the resin matrix containing 0.01 wt% of fluorescein (Sigma-Aldrich, Milwaukee WI, USA). One drop of the material was dispensed in a FluoroDish (Fisher Scientific, Waltham, MA, USA), forming a sample 650 µm thick. The samples were imaged using an inverted confocal laser scanning microscope (ZEISS LSM 880, CarlZeiss US, White Plains, NY, USA) with a 63 × 1.4NA objective and the Fast Airyscan module^17^. Fast Airyscan increases acquisition speed and improves resolution. Increased speed is achieved by modulating the laser beam from round to oblong, which can scan four pixels at once. Improved resolution is achieved by capturing 16 images of a single field of view across a specialized detector. Each image contains positional information that enables the reconstruction of a single super-resolution image^17^. Samples were excited at 561 nm and 488 nm. Z-stacks were collected with a 0.2 µm step size over 11.25 µm. The micrographs were processed using the ZEN 3.1 Blue and Black editions software. Thiourethane coating thickness was measured in 2D images at the central Z-stack slice of the sample using the software Zeiss ZEN 3.1 (blue edition) (Carl Zeiss Microscopy GmbH). Three lines (horizontal, vertical, and diagonal) were drawn along the image of representative filler particles and the average of the six points is reported.

### Statistical analysis

Data were statistically analyzed with three-way ANOVA and Tukey’s test for multiple comparisons (α = 0.05) after verifying normality and homoscedasticity (Anderson–Darling and Levene tests, respectively). First, a three-way ANOVA was conducted to determine possible interaction between the different factors (filler amount, size and surface treatment). Then, to facilitate data interpretation, each of the filler particle sizes was analyzed separately using two-way ANOVA and Tukey’s test (α = 0.05).

## Results

**Figure 2:**
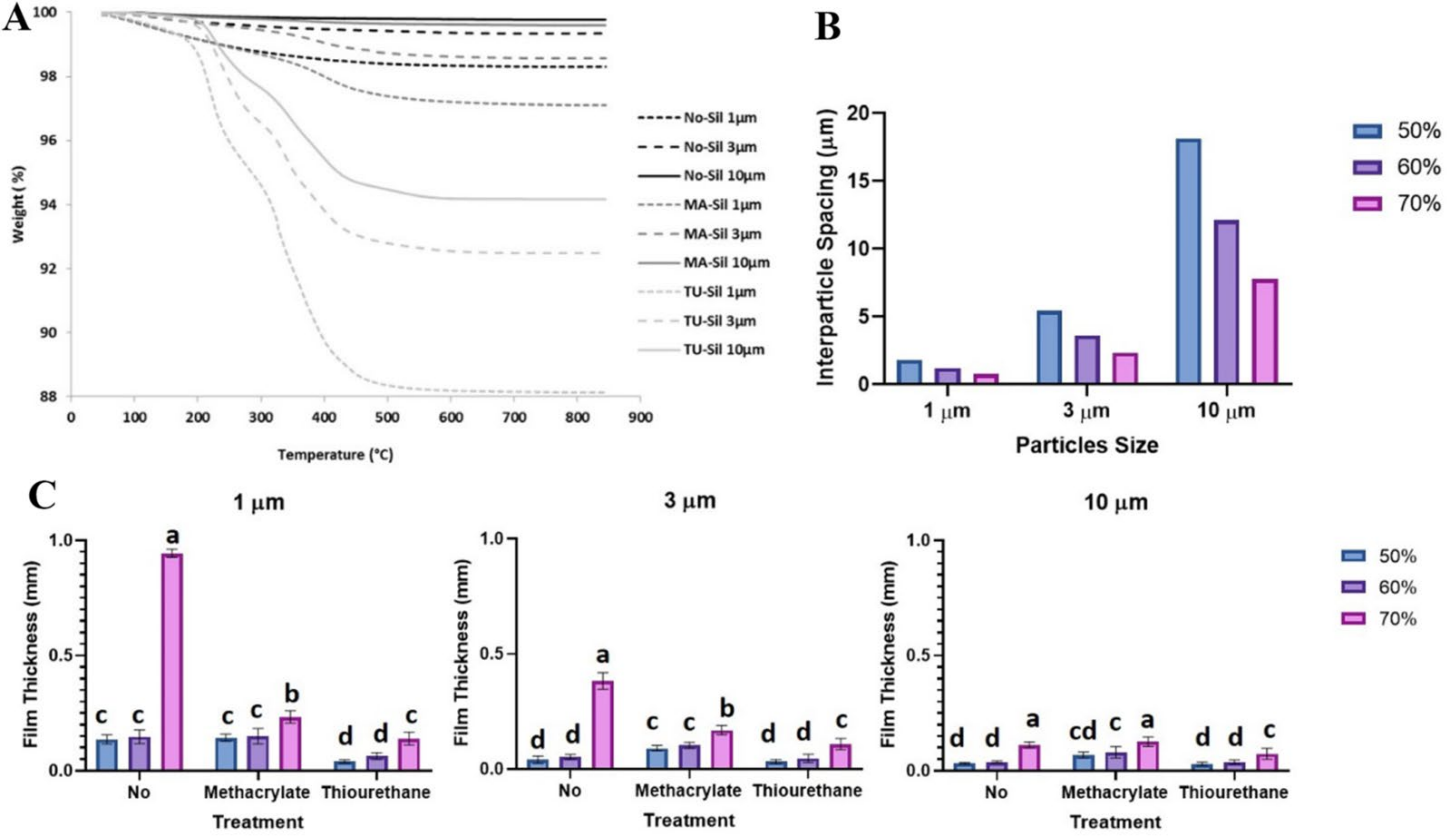
(**A**) Thermogravimetric curves displaying weight loss (%) as a function of temperature (°C) for each filler particle. (**B**) Interparticle spacing as a function of particle size (µm) and filler percentage by volume (vol%). (**C**) Film thickness (µm) for all tested groups. Different letters indicate statistically significant differences between the groups with the same particle size (p < 0.05).

The TGA results show that filler surface coverage ranged between 11.7 and 5.8% for thiourethane-functionalized particles and between 2.9 and 0.4% for methacrylate-silanized fillers (Fig. 2). Unsilanized particles showed a mass ranging between 0.1–0.8% during the experiment, likely attributed to water adsorption by these more hydrophilic particles^18^. In general, TU fillers had 4–5 times greater mass loss than MA particles, and the weight loss increased with the decrease in filler size. Inter-particle spacing distance was calculated assuming spherical particles and lack of agglomeration, and is presented along with the results for clarity. As expected, spacing increased with the increase in filler size at each filler volume (Fig. 2B).

Surface functionalization and particle size played a significant role in all experimental tests (p > 0.05). Filler particle percentage did not affect DC at RP_MAX_ and final DC (p = 0.121 and 0.615, respectively). The three-factor interaction was significant, except for DC at RP_MAX_ and final DC (p = 0.325 and 0.074, respectively). Data for each of the filler particle sizes was analyzed separately by two-way ANOVA and Tukey’s test (Table 1). Three-way ANOVA for all tested factors and interactions are also shown in Table 1.

**Table 1:** Three and two-way ANOVA partitions (p values).

Three-way	Tests	Surface treatment	Size	% Filler	Surface treatment * size	Size * % filler	Surface treatment * % filler			
	RP_MAX_	< 0.0001	< 0.0001	0.002	< 0.0001	0.001	< 0.0001			
	DC at RP_MAX_	< 0.0001	< 0.0001	0.121	< 0.0001	0.006	0.141			
	Final DC	0.004	< 0.0001	0.615	< 0.0001	0.240	0.208			
	KIC	< 0.0001	< 0.0001	< 0.0001	0.002	0.032	< 0.0001			
	Stress	< 0.0001	< 0.0001	< 0.0001	0.130	0.051	0.002			
	Film Thickness	< 0.0001	< 0.0001	< 0.0001	< 0.0001	< 0.0001	< 0.0001			

Two-Way	Tests	1 µm			3 µm			10 µm		
		% Filler	Surface treatment	Interaction	% Filler	Surface Treatment	Interaction	% Filler	Surface treatment	Interaction
	RP_MAX_	0.0712	< 0.0001	0.0954	0.0019	< 0.0001	< 0.0001	0.0217	< 0.0001	0.0062
	DC at RP_MAX_	0.7527	< 0.0001	0.4859	0.0009	< 0.0001	0.3954	0.8816	< 0.0001	0.2531
	Final DC	0.0275	< 0.0001	0.0692	0.0863	< 0.0001	0.0393	0.0087	< 0.0001	0.0140
	KIC	0.0030	< 0.0001	0.2321	0.1658	< 0.0001	0.2432	0.0527	< 0.0001	0.8559
	Stress	< 0.0001	< 0.0001	0.0028	0.0727	< 0.0001	0.2474	0.0799	< 0.0001	0.0003
	Film Thickness	< 0.0001	< 0.0001	< 0.0001	< 0.0001	< 0.0001	< 0.0001	< 0.0001	< 0.0001	0.0534

Three-way ANOVA analyzed the three variables and their interaction in all experiments. Two-way ANOVA considered the effect of the filler amount, particle surface treatment and interaction between the two factors for each filler particle size in all experiments. The significance level was α = 0.05for both analyses.

**Figure 3:**
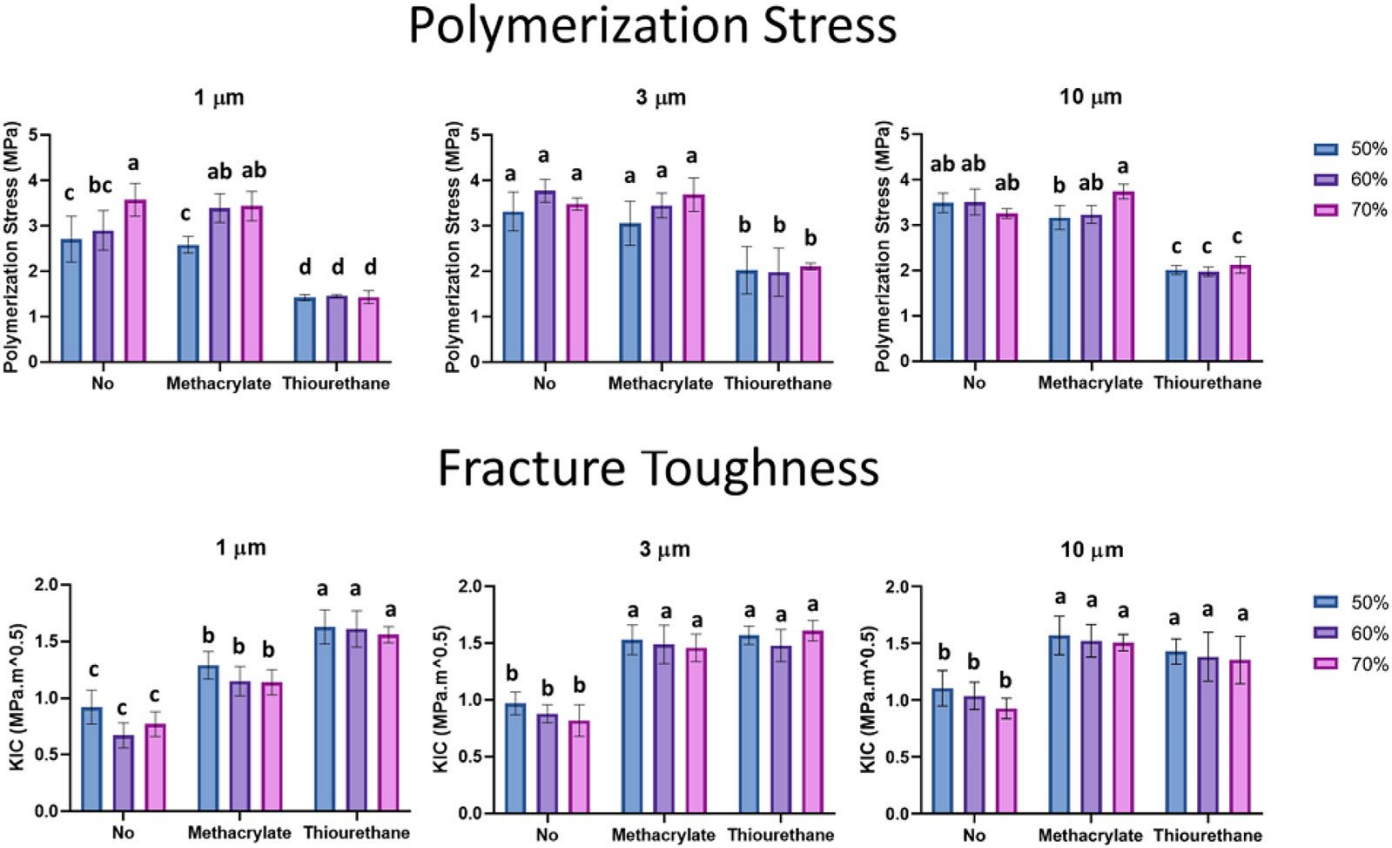
Polymerization stress (PS, MPa) and fracture toughness (KIC, MPa^.^m ^0.5^) for all groups. Different letters indicate statistically significant differences between the groups with the same particle size (p < 0.05).

TU-Sil groups showed significantly lower polymerization stress in comparison with No-Sil and MA-Sil groups: 41% for 3 and 10 µm filler size, and 54% for 1 µm-sized particles. Stress was not affected by the filler percentage within the same type of surface treatment, with only a few exceptions (No-Sil 1 µm; MA-Sil 1 µm; MA-Sil 10 µm, where the stress was higher with the 70% filler in comparison with the 50%). No-Sil and MA-Sil exhibited comparable results regardless of the particle size and filler volume (p > 0.05). The polymerization stress was significantly affected by all tested factors, but the interaction between surface treatment *versus* particle size, and particle size *versus* filler percentage were not significant (p > 0.05) (Table 1 and Fig. 3).

For fracture toughness, all three tested factors and interactions were statistically significant (p < 0.05) (Table 1). TU-Sil groups presented the highest values for 1 µm-sized filled composites, ranging between 1.63 and 1.56 MPa.m^0.5^ (Fig. 3). For 3 and 10 µm particles, there was no statistical difference between TU-Sil and MA-SIL groups (p > 0.05). No-Sil groups showed the lowest results regardless of the tested particle size. For all surface treatments and particles sizes, the filler percentage did not affect the fracture toughness results (p > 0.05).

**Figure 4:**
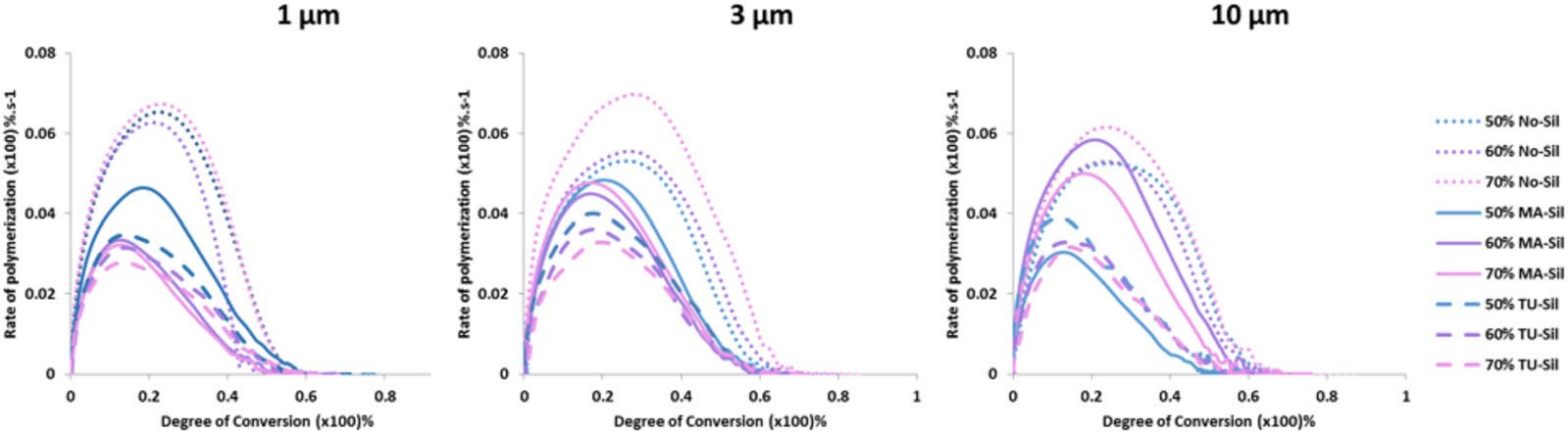
Rate of polymerization (%.s^−1^) as a function of degree of conversion (%) for experimental resin composites containing different filler particle contents. Vinyl conversion was followed in real-time by near-IR for 180 s.

TU-Sil showed the highest values of final DC (ranging between 71.5 and 68.3%) and MA-Sil particles the lowest ones (ranging between 63.7 and 53.8%) for filler particles of 1 and 3 µm. For 10 µm particles, MA-Sil showed the highest final DC values (ranging bewteen 70.8 and 72.2%) and TU-Sil the lowest ones (ranging between 59.2 and 61.6%). In terms of kinetics of polymerization, in general, No-Sil groups had higher RP_MAX_ and DC at RP_MAX_, regardless of the filler particle size (Fig. 4 and Table 2). TU-Sil groups showed similar or lower results, and were comparable to MA-Sil groups.

**Table 2:** Average ± SD of maximum rate of polymerization (RP_MAX_, %.s^−1^), degree of conversion at maximum rate of polymerization (DC at RP_MAX,_ %), and final degree of conversion (Final DC, %).

	Groups	1 µm			3 µm			10 µm		
		50%	60%	70%	50%	60%	70%	50%	60%	70%
RP_MAX_	No-Sil	6.5 ± 0.4^**A**^	6.3 ± 0.1^**A**^	6.7 ± 0.1^**A**^	5.3 ± 0.1^**BC**^	5.7 ± 0.1^**B**^	7.9 ± 0.4^**A**^	5.5 ± 0.5^**ABC**^	5.2 ± 0.5^**ABC**^	6.3 ± 0.2^**A**^
	MA-Sil	3.5 ± 0.8^**B**^	3.2 ± 0.1^**B**^	3.1 ± 0.1^**B**^	4.5 ± 0.1^**CDE**^	4.8 ± 0.2^**BCD**^	4.7 ± 0.5^**CD**^	5.8 ± 0.4^**AB**^	5.0 ± 0.3^**C**^	4.8 ± 0.2^**C**^
	TU-Sil	3.5 ± 0.4^**B**^	3.1 ± 0.1^**B**^	2.8 ± 0.2^**B**^	4.5 ± 0.6^**CDE**^	3.6 ± 0.4^**EF**^	3.3 ± 0.4^**F**^	3.9 ± 0.4^**D**^	3.4 ± 0.4^**D**^	3.2 ± 0.3^**D**^
DC at RP_MAX_	No-Sil	21.9 ± 0.5^A^	21.7 ± 1.2^A^	22.7 ± 1.5^A^	24.9 ± 2.5^AB^	27.4 ± 1.1^A^	27.9 ± 1.4^A^	26.3 ± 0.6^A^	25.2 ± 1.2^A^	23.0 ± 5.0^A^
	MA-Sil	12.9 ± 1.6^**B**^	12.4 ± 0.4^**B**^	11.6 ± 1.6^**B**^	16.7 ± 0.5^**CD**^	17.3 ± 1.6^**CD**^	19.0 ± 1.4^**CD**^	20.9 ± 1.1^**AB**^	19.5 ± 1.4^**AB**^	20.4 ± 0.3^**AB**^
	TU-Sil	14.0 ± 0.2^**B**^	13.9 ± 0.7^**B**^	13.4 ± 0.7^**B**^	15.0 ± 0.6^**D**^	18.1 ± 1.9^**CD**^	20.9 ± 2.1^**BC**^	12.1 ± 2.6^**C**^	15.3 ± 3.4^**BC**^	14.8 ± 2.0^**BC**^
Final DC	No-Sil	63.4 ± 4.7^B^	62.4 ± 5.0^BC^	57.2 ± 1.0^CD^	66.5 ± 0.8^BC^	70.4 ± 2.9^AB^	71.7 ± 1.0^A^	64.1 ± 0.7^C^	64.2 ± 0.8^C^	67.8 ± 0.6^B^
	MA-Sil	55.1 ± 3.1^D^	54.6 ± 0.7^D^	53.8 ± 1.3^D^	62.3 ± 0.6^C^	63.4 ± 2.1^C^	63.7 ± 1.5^C^	72.8 ± 0.2^A^	70.8 ± 0.4^A^	71.5 ± 0.3^A^
	TU-Sil	68.4 ± 0.2^A^	68.3 ± 0.5^A^	68.4 ± 0.2^A^	71.5 ± 1.0^A^	70.8 ± 0.9^AB^	70.0 ± 1.5^AB^	59.2 ± 2.6^D^	60.5 ± 0.1^D^	61.6 ± 1.2^CD^

Regarding film thickness, all studied factors and interactions were statistically significant (p < 0.05, Table 1 and Fig. 2C). 70%-filled groups produced the highest film thickness regardless of the surface treatment and particle size. Film thickness was highest at 1 µm and 3 µm for the composites with 70% fillers with no treatment. No difference was observed between 50 and 60%-filled groups.

**Figure 5:**
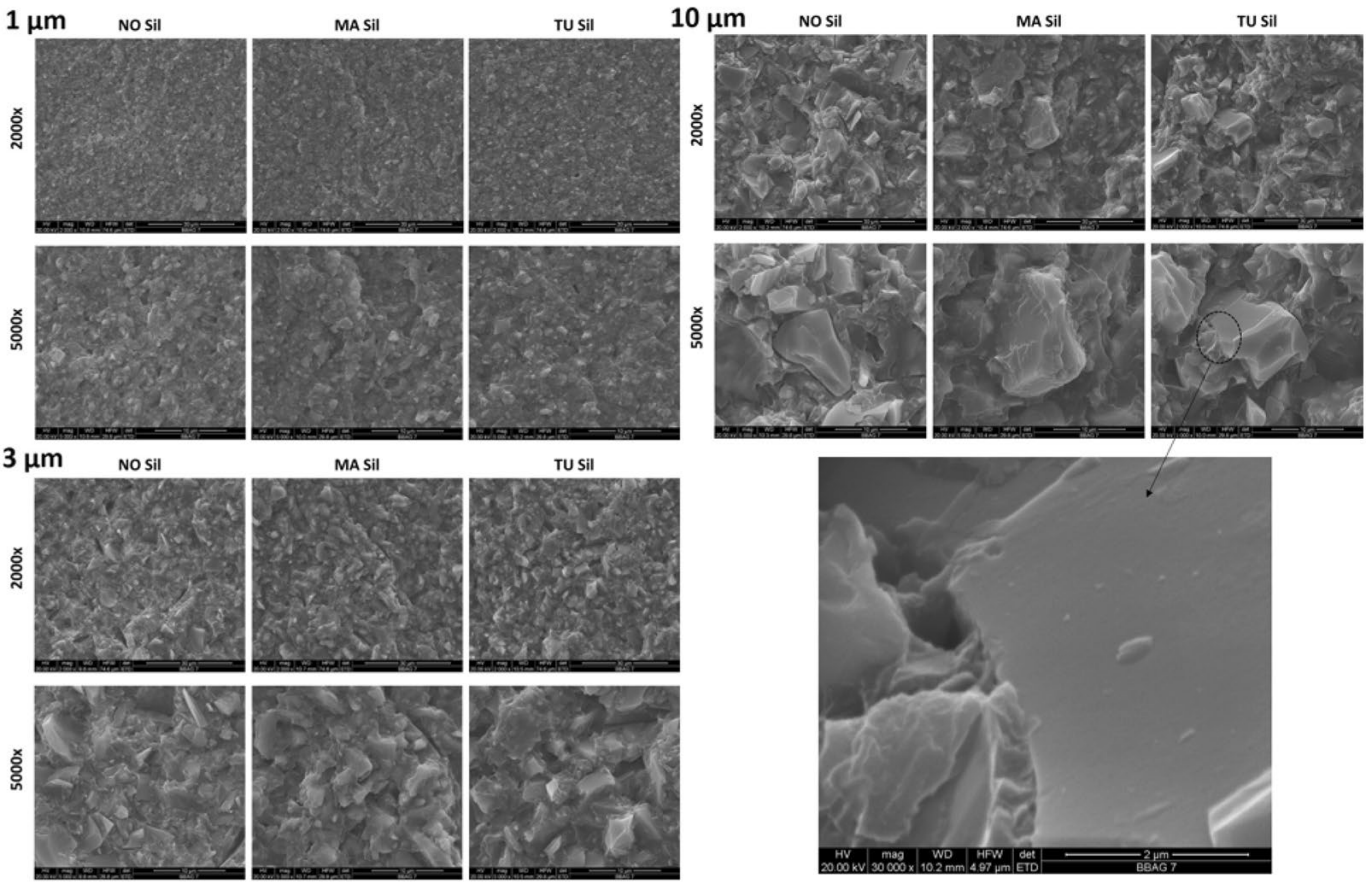
SEM micrographs of fractured surfaces of fracture toughness bars filled with 70 wt% filler particles of 1 µm, 3 µm, and 10 µm size under 2000 × and 5000 × magnifications. In general, NO-Sil formulations were characterized predominantly by uncoated particles completely disconnected from the organic matrix with several areas indicating detachment of particles. In MA-Sil systems most particles are evenly coated by the organic matrix. TU-Sil groups show intermediate characteristics, with particles that were completely uncoated and others seemingly intimately bonded to the organic matrix, as highlighted in micrograph under 30,000 × magnification. It also appears that the TU-Sil fractured surfaces are more distinct, and less “blurry”, unlike in the MA where the crack seems to propagate through the matrix predominantly with the filler well bonded to the resin. The TU shows less bonding in comparison.

The analysis of fractured surfaces from fracture toughness bars showed marked differences in terms of filler-organic matrix interface among the three surface treatments (Fig. 5). NO-Sil groups showed bare particles clearly separated from the organic matrix, most easily visualized for the largest particle size. MA-Sil typically showed particles entirely encapsulated within resin matrix, and TU-Sil particles presented a mixed aspect (bare and covered particles), with the exposure of filler surface most obvious for the two larger filler sizes.

**Figure 6:**
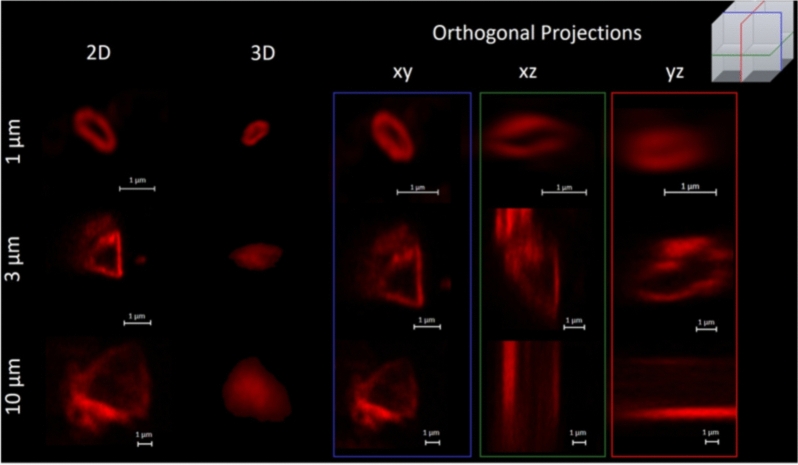
2D view, 3D top view, and x–y, x–z, and y–z orthogonal projections of confocal section of individual rhodamine-tagged thiourethane functionalized filler particles incorporated in BUT organic matrix. The confocal images show an irregular shaped filler particle covered by an oligomeric layer (tagged in red), averaging 649 nm, 1226 nm and 1885 nm in thickness for the 1, 3 and 10 µm particles, respectively. The orthogonal projections show uneven thiourethane coating along the particle surface, with alternating regions showing a layer of up to 600 nm thickness, and other regions with no coverage (bare particle). Note: the orthogonal projections represent the cross-sections of particles, as indicated in the color-coded insert on the top right corner of the figure. To keep the size bars consistent across different projections, the 3 and 10 µm particles are not shown in their entirety in the xz and yz projections.

The confocal fluorescent images for the TU-containing groups are presented in Fig. 6. The methacrylate silane groups were not imaged with this technique because the coating’s minimal thickness fell below the resolution of the confocal microscope. In Fig. 6, individual particles are shown in three different perspectives: 2D, 3D top view and x–y, x–z, and y–z orthogonal projections of a confocal section. The projection images show large regions with no coverage, especially for the 10 µm-sized filler particles. The average coating thickness of the filler particles were measured in the 2D images using the histogram feature on Zen Blue (Zeiss) as an average of 6 different regions. The thickness for the 1, 3 and 10 µm particles was 649 ± 107 nm, 1226 ± 334 nm, and 1885 ± 981 nm, respectively.

## Discussion

The incorporation of pre-polymerized additives, including thiourethane oligomers, to the formulation of resin composites has shown desirable outcomes, such as the reduction in volumetric shrinkage^19^ and shrinkage stress^4,19^, and increase in fracture toughness^4–6^. However, this strategy usually leads to a non-negligible increase in viscosity, which prevents the incorporation of higher percentages of inorganic fillers^6^, and negatively affects handling characteristics. This ultimately limits the amount of pre-polymers that can be added before other properties start to deteriorate (^6^—30% TU in cements). For this reason, the functionalization of the inorganic filler surface with oligomeric species has been proposed, and, at least for thiourethanes, has shown similar reduction in polymerization stress and improvement in mechanical properties without compromising the viscosity of the composite paste^7,8^. In this study, TU functionalized filler particle characteristics were studied in a systematic fashion to gain insight into the mechanisms leading to the reinforcing and stress-reducing outcomes observed with thiourethane oligomers.

The same standard functionalization method was used for the thiourethane and the conventional methacrylate silane (MA-Sil). The grafting to the filler surface was accomplished in a slightly acidic aqueous alcohol solution (pH ≅ 4.5) to hydrolyze the alkoxy groups, forming reactive silanols (≡Si–OH), which further react with themselves by a condensation process to form oligomeric siloxanes (–Si–O–Si–bonds). At the same time, the growing silane networks link to the inorganic substrate through hydrogen bonds with the hydroxyl groups present on the particle surface^20–22^. The filler surface functionalization efficiency was assessed by thermogravimetric analysis (Fig. 2A). For the particles functionalized with the MA-Sil, all organics were burned off at 500 °C and no additional mass was lost at higher temperatures. For TU-Sil fillers, the maximum mass loss was observed closer to 600 °C, which was also expected based on the much greater molecular weight of the oligomer compared to the small-molecule methacrylate silane. The untreated particles (NO-Sil), used as received from the manufacturer, also showed mass loss at a similar temperature, which could be due to impurities from the manufacturing process. For all surface treatments, the mass loss increased with a decrease in the average particle size^23^, which was expected since at the same mass, 1 µm-sized particles have ten times more free surface area available to be functionalized than 10 µm-sized particles (Table 3). It is noteworthy that the mass loss as a function of surface area was different for each surface treatment. For the NO-Sil and MA-Sil groups, 1 µm-sized particles showed ten times more weight loss than 10 µm-sized particles: 1.61 and 0.22% for 1 µm and 10 µm-sized particles, respectively (NO-Sil) and 2.91 and 0.39% for 1 µm and 10 µm-sized particles, respectively (MA-Sil). For the TU-Sil groups, the mass loss did not linearly scale with the particle size (weight loss = 11.73% and 5.82% for 1 µm and 10 µm-sized particles, respectively). This difference can be at least partially explained by differences in molecular weight (~ 250 g/mol for the MA-Sil and 5 kDa—or roughly 5,000 g/mol—for TU-Sil)^6^, as well as differential thickness of the coating. The monotonic increase in weight loss with decreasing filler size is consistent with the formation of a monolayer for the methacrylate silanes. In contrast, it is possible that the thiourethane oligomer is crosslinked onto the surface, which will be further discussed later. It is also possible that the silanization process with the thiourethane oligomer is not as efficient, and that results in non-uniform functionalization, as shown in the imaging results.

**Table 3:** Surface area (µm^2^), volume (µm^3^), and mass (g) for each size of the particles, considering the filler particle density 2.5 g/cm^3^ and the shape being a sphere.

Particle size	Particle surface area (S_Area_ = 4πr^2^) (µm^2^)	Particle volume (V = 4/3 πr^3^) (µm^3^)	Particle mass (considering d = 2.5 g/cm^3^) (g)	Number of particles in 5 g (× 10^12^)	Free surface area in 5 g of particles (× 10^12^ µm^2^)	Mass loss (from TGA) in %—for TU-Sil	Surface thickness from confocal (nm) – for TU-Sil
1 µm	3.14	0.52	1.3	3.84	12.0	12	649
3 µm	28.26	14.13	35.3	0.14	3.95	7.5	1226
10 µm	314	523	1307	0.00384	1.20	6	1885

The mass was randomly fixed as 5 g, since the aim was to highlight differences in free surface area of the particles subjected to the functionalization procedures.

**Figure 7:**
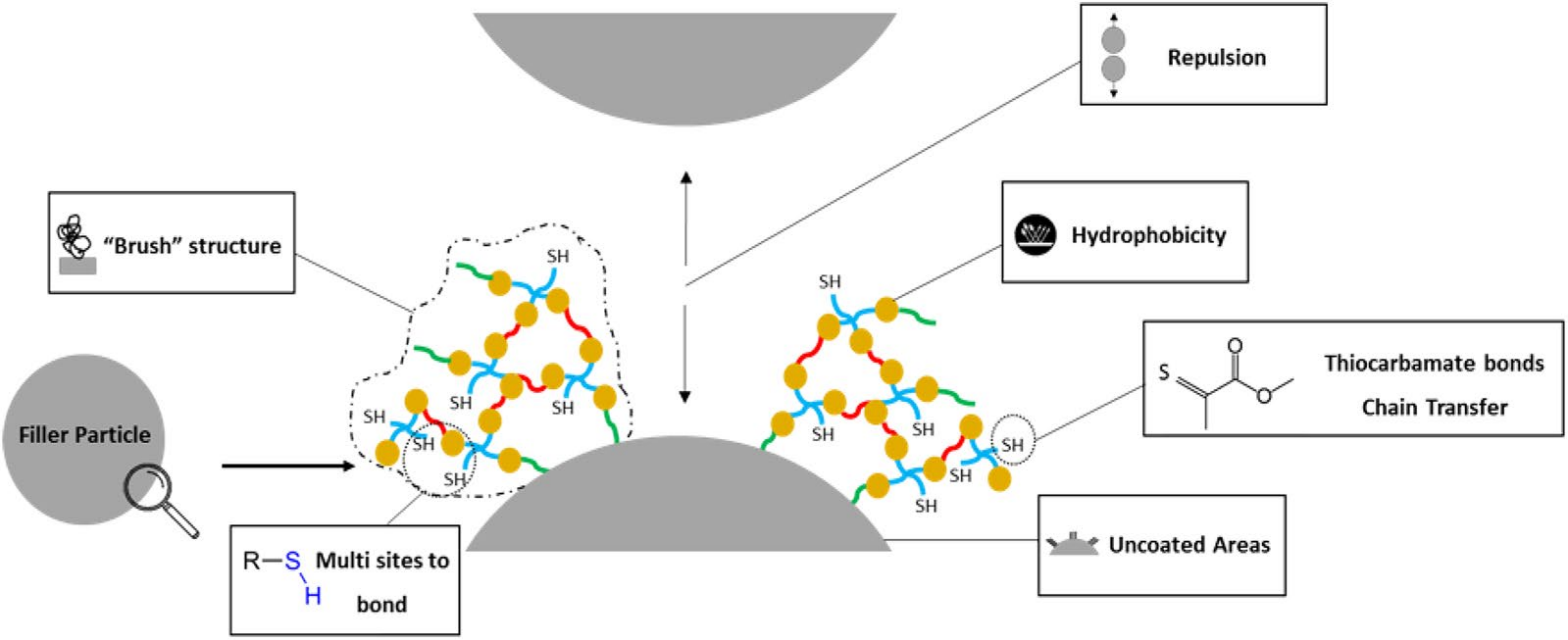
Schematic representation summarizing the multiple mechanisms potentially involved in the stress reduction and the toughness increase for composites containing inorganic filler particles functionalized with thiourethane oligomers.

SEM micrographs (Fig. 5B) were used to characterize the particle–matrix interface. While the MA-Sil particles are entirely coated with organic matrix, thiourethane particles seem to be more heterogeneously coated, with some naked regions, and others showing better interaction with the matrix (Fig. 5C). This pattern is also identified in the confocal images which show uneven distribution of the thiourethane oligomer along the filler particle surface (Fig. 7), alternating areas with a layer of 649–1885 nm in thickness depending on the filler size and other naked regions (Table 3 and Fig. 6). The coating on the larger filler particles was much more disperse, with extensive areas of the filler remaining uncovered. The bulky thiourethane oligomer is likely tethered onto the filler surface at multiple locations, creating steric constraints for additional chain grafting, ultimately limiting the overall graft density^24^. It is important to point out that the starting concentration of TU oligomer during the filler functionalization was the same for all filler sizes, and the mass of filler used here was the same regardless of the size. This results in much greater overall surface area and smaller inter-particle distancing in the smaller filler particles. This likely explains why the thickness of the silane layer was similar for the 1 and 3 µm particles, at around 649 and 1226 nm, respectively, but much thicker for the 10 µm particles (1885 nm). In summary, for the larger particles, the greater inter-particle spacing and smaller overall surface area, likely combined to increase the coating thickness. The surface area vs. particle size relationship also explains the mass loss data from the TGA, which shows greater mass loss with the smaller particles, again due to the greater filler surface area per volume of material afforded by smaller particles. In addition to the mechanisms that will be discussed in more detail later, it is possible that the uncoated areas act as defect sites, which are known to lead to lower stress development^25^.

During polymerization, the monofunctional methacrylate silane co-polymerizes with the vinyl-containing matrix, and establishes a short and rigid bond with the filler surface^20^, which contributes to stress generation. In contrast, the high molecular weight thiourethane silane establishes multiple covalent interactions with the polymerizing organic matrix via the pendant thiols. The bonds formed are flexible thiocarbamate bonds which can serve as sites for relaxation of the overall polymerization stress^26^. In the specific case of the thiuorethane oligomers studied here, some stress relaxation during polymerization is also afforded by the delayed gelation vitrification^5–7^, but more recent studies have also pointed to the possibility of stress relaxation via dynamic bond adaptation behavior in the glassy state^27–30^. The dynamic relaxation behavior of thiourethanes is currently being investigated and will be reported separately. Preliminary studies using time–temperature superposition experiments have already demonstrated faster relaxation times for fillers treated with TU-Sil^31^. Different from the strategy where the stress-relieving molecules is randomly distributed in the matrix^4–6^, in composites with TU functionalized fillers, the stress relieving molecules are localized at the filler-matrix interface, a region of stress concentration^32^. This may account for the significant reduction in stress transfer between the two constituent phases of the resin composites^25^, even at a much lower overall TU concentration than that present when TU is added directly to the resin matrix. Additionally, the thiourethane likely forms thick and highly dense polymer structures on the surface of the particle, which may also contribute to dissipate part of the generated stress^33,34^ and to increase plastic deformation during stress development that help accommodate changes in free volume^34,35^. The multifunctional nature of thiourethanes also leads to differences in crosslinking in the siloxane layer and enhanced interface adhesion^25^. This may compensate for the uneven coverage of the filler particle by the thiourethane silane mentioned earlier, and helps explain why mechanical properties are not compromised, in spite of the presence of naked regions on the filler particle surface. Finally, the stress development and the mechanical properties of the resin composites are also dependent on the degree of agglomeration of the filler particles^36^. Systems with greater filler dispersion show improved storage modulus, tensile strength, toughness and lower polymerization stress due to the enhanced matrix-filler interaction and interfacial adhesion^25^. When polymer chains are grafted onto the particle surface there is steric repulsion, which minimizes their tendency to agglomerate due to the van der Walls attraction and, ultimately, promotes a more uniform dispersion of the particles.

The stress development can also be correlated with the kinetics of polymerization. The fact that with NO-Sil and MA-Sil composites had similar stress behavior was unexpected since, in theory, in the absence of bonding between the particles and the organic matrix, the filler particles should effectively behave as voids, leading to stress relief^37^. However, in general NO-Sil composites showed markedly faster polymerization reaction, which may have led to earlier development of diffusional limitations and a rise in stiffness, minimizing the opportunity for stress relaxation. The higher RP_MAX_ and DC at RP_MAX_ found in NO-Sil formulations may have resulted from the increased system mobility imposed by the absence of functional silanes at the interface^38^. As expected, the addition of TU-Sil led to significant reduction in polymerization stress, ranging between 41 and 54% in comparison with the MA-Sil groups. The reduction in stress is explained by chain-transfer reactions of the thiols with the vinyl groups, which delays the point in conversion at which the stiffness of the networks begins to significantly increase, and past which any increase in conversion results in disproportionately higher stress. In general, the addition of TU-Sil particles into the resin composite formulations decreased the RP_MAX_, as well as the conversion registered at RP_MAX_, in agreement with previous studies^4^, indicating at least some effect in delayed network formation (Fig. 4). The somewhat slower polymerization reaction did not compromise the final DC; on the contrary, TU-Sil formulations showed the highest values for 1 and 3 µm-sized filler particles. For the 10 µm-sized particles, conversion was similar to the methacrylate controls. This may be due to the lower concentrations of thiourethane in this composition (Table 4), which may have been insufficient to affect the polymerization reaction kinetics. Chain-transfer reactions are also responsible for more homogeneous network formation, which decreases the development of internal stress, especially immediately after the diffusion limitation occurs^5,39,40^. It is also possible that the simple presence of the low Tg thiourethane on the surface of the filler particle may play a similar role in stress relief, acting as a ductile zone for plastic deformation between the filler and the organic matrix, which ultimately yields stress absorption at the interface and toughening. Interestingly, it seems that the localization of thiourethanes directly at the surface of the filler particle significantly decreases the overall concentration of thiourethane needed to produce significant reduction in polymerization stress, compared to what is needed when TU is added directly into the matrix^4–6^. Therefore, several mechanisms may be operating at the filler-matrix interface, as mentioned throughout the discussion, and summarized in Fig. 7. It is important to reiterate, however, that the stress reduction is not gained at the expense of reduced conversion.

**Table 4:** Percentage of thiourethane (%) in each TU-Sil resin composite group considering the filler load and the variation in filler surface coverage obtained from the TGA results and the estimated threshold based on previous literature^5^.

Filler wt%	1 µm	3 µm	10 µm	Estimated threshold
50	5.87%	3.85%	2.91%	10%
60	7.04%	4.62%	3.50%	8%
70	8.21%	5.39%	4.08%	6%

The data show that the replacement of 20 wt% of the organic matrix by thiourethane oligomer is the optimized concentration in order to decrease the polymerization stress without compromising the elastic modulus.

A potential concern over the uneven coverage of the filler particles with the TU might be the susceptibility of the particles to being dislodged under load, consequently, compromising the mechanical properties. In this study, the fracture toughness results showed the opposite effect for the 1 µm TU-Sil, with significant enhancement for all the three groups containing TU-treated particles (1.60 ± 0.03 MPa•m^1/2^) in comparison to MA-Sil groups (1.19 ± 0.08 MPa•m^1/2^) (Fig. 3). This 34% increase in fracture toughness is attributed to the flexible thiocarbamate covalent bonds^4^, and may also be due to lower levels of internal stress accumulation^41,42^. For 3 and 10 µm-sized fillers, there were no statistical differences between TU-Sil and MA-Sil, which indicates that the percentage of the thiourethane incorporated into the mixtures (Table 5) was not sufficient to significantly improve the fracture toughness. In fact, the confocal images demonstrate that the TU-Sil layer was much more uneven than for the 1 µm filler, which correlates with the data for mass loss shown in Table 3, as already mentioned. The SEM micrographs of the fracture surfaces containing TU-Sil particles showed chunks of organic matrix covering some regions of the particle’s surface, which may indicate that the fracture sometimes propagated through the organic matrix, but also along the resin-filler interface in these systems. In contrast, in the MA-Sil systems the fractured surfaces were typically covered by a thin and uniform layer of resin, which indicates that the fracture propagated through resin matrix near the fillers. This adds evidence to the fact that, though not being entirely coated by the silane, the interaction of the inorganic fillers to the organic resin in thiourethane-containing systems still allows for lower stress concentration at the filler-matrix interface. In addition, the uncoated areas on the TU-Sil filler particles might provide an energy releasing path around the particles, contributing to the enhanced toughness^37^. Groups containing NO-Sil particles showed, as expected, the lowest fracture toughness, due to the absence of interfacial bonding between the filler particles and the organic matrix. The mass percentage of filler particles incorporated into the formulation did not impact the mechanical resistance, which is in agreement with results reported previously in the literature for composites with different levels of 1 µm-sized barium-alumina borosilicate particles^43^. This previous study has shown that the flexural strength decreases and the flexural modulus increases slightly as the filler particle load increases from 40 to 60 wt% and, above 60 wt%, there is a gradual increase in both flexural strength and modulus. The results were correlated with particle size distribution, particle–matrix adhesion strength, and arrangement of the filler particles into the organic matrix^43^. In highly loaded systems (above 60 wt%), there was a tendency for a percolated network particle structure to be formed, which are aggregates of filler associated with mechanical reinforcement^43^. However, it is important to highlight that there is a threshold for increase in filler particle content and increase in mechanical performance, above which the addition of higher amounts of filler leads to decreased particle–matrix adhesion strength, possibly due to the formation of agglomerates. This threshold varies according to the filler particle system. It is possible to assume that, at least for the larger filler sizes (3 and 10 µm), the load range used in this study was insufficient to result in significant differences in fracture toughness. For the 1 µm filler particles, the fracture toughness results agree with our previous work demonstrating significant increase in values when comparing methacrylate vs thiourethane silanes^7^.

**Table 5:** Actual filler particles percentage by weight (wt %) and by volume (vol %) for each tested experimental composite considering the variation in filler surface coverage showed by TGA results.

Groups		1 µm			3 µm			10 µm		
		No-Sil	MA-Sil	TU-Sil	No-Sil	MA-Sil	TU-Sil	No-Sil	MA-Sil	TU-Sil
wt%	50	49.19	48.54	44.13	49.71	49.36	46.15	49.89	49.80	47.09
	60	59.03	58.25	52.96	59.65	59.23	55.38	59.86	59.76	56.50
	70	68.87	67.96	61.79	69.60	69.11	64.61	69.84	69.72	65.92
vol%	26.8	26.23	25.73	22.48	26.63	26.36	23.94	26.77	26.70	24.63
	35.5	34.60	33.88	29.25	35.19	34.79	31.31	35.38	35.29	32.29
	46.2	44.82	43.79	37.26	46.67	45.10	40.13	45.96	45.81	41.53

One thing to note is that, even though the filler loading followed standardized mass ratios (50, 60, and 70 wt%), for the TU-Sil groups, a much higher percentage of the filler weight corresponded to the mass of the silane, compared to the methacrylate groups. As a consequence, the actual inorganic filler loading varied significantly among the groups (Table 5). In general, TU-Sil composites contain 10 wt% less filler than MA-Sil formulations with 1 µm-sized particles, 6.5 wt% less with 3 µm-sized particles, and 5.4 wt% less with 10 µm-sized particles. This translated into thinner film thickness for groups containing TU-Sil particles (Fig. 2C). In addition, the film thickness is also affected by the distance between particles. For smaller filler sizes, the particles become more compacted (closer to each other), which also leads to an increase in filler content (Fig. 2B). As expected, the smaller particles led to greater viscosity as more of the resin matrix is influenced by contact with the fillers, and the smaller inter-particle spacing leads to more filler-filler interactions, both of which result in thickening of the paste.

An additional potential advantage of the thiourethane coating is its hydrophobicity. As it is well known, the presence of ester bonds makes the methacrylate silane prone to hydrolytic degradation. The siloxane layer degradation, caused by the vulnerability of the oxane bonds to hydrolysis due to its significant ionic character, increases the concentration of hydroxyl ions^44^, which leads to an autocatalytic reaction. As result of this reaction, there is a weakening of the filler-matrix bonding, leaching of chemical compounds, generation of micro-cracks at the interface, particle debonding, and, ultimately compromised mechanical properties^45^. Even though it was not the main goal of the present study to investigate the hydrolytic stability of the interfaces, the use of the thiourethanes (a multifunctional, hydrophobic, crosslinked, high molecular weight oligomer) as a particle coating can be envisioned to improve the durability and the hydrolytic stability of the interfacial siloxane bond. This may ultimately improve the durability of the filler-matrix bonding.

## Conclusion

In general, the functionalization of the inorganic filler particles surface with the prepolymerized thiourethane significantly reduced polymerization stress generation without compromising the viscosity and film thickness of the materials. In addition, a significant enhancement in fracture toughness was observed in thiourethane formulations filled with 1 µm-sized particles. The stress reducing/relieving and toughening effects of the polymeric network, however, have been confirmed with the use of the thiourethanes in this filler-functionalization strategy, even though several concomitant mechanisms are likely at play.
